# Heart or head?: A depression mimic delays diagnosis—oncotic cerebral aneurysms caused by atrial myxoma

**DOI:** 10.1259/bjrcr.20170028

**Published:** 2017-06-22

**Authors:** Bridgid Connolly, Simon J Prowse, Catherine E Connolly, Nicholas J Brett

**Affiliations:** ^1^Radiology Department, Flinders Medical Centre, Adelaide, South Australia; ^2^Department of Radiology, Royal Adelaide Hospital, Adelaide, South Australia

## Abstract

Complications of intracardiac tumours can carry significant morbidity and mortality. This article depicts the case of a female who presented with multiple oncotic intracranial aneurysms secondary to a left atrial myxoma. The clinical manifestations and pathogenesis of cardiac myxoma, as well as the imaging pathway, management and prognosis of myxomatous aneurysms will be discussed. Excision of the cardiac mass is mandatory both for symptomatic improvement and to prevent further embolic complications. Local recurrence and delayed onset cerebral complications do occur, and necessitate ongoing patient follow-up. Our report highlights several important features of the diagnostic and treatment pathway for atrial myxoma—in particular, the potentially non-specific clinical presentation, the pivotal role of cardiac MRI in the multimodality diagnostic imaging work up and the need for multidisciplinary communication to identify the diagnosis and guide appropriate management.

## Background

Neurologic complications of intracardiac tumours can carry significant morbidity and mortality. Despite the potential for serious or life-threatening sequelae, the rarity of myxomas means the pathway from presentation to final diagnosis can be circuitous and prolonged. One case series reported a median delay from the onset of neurological symptoms to diagnosis of cardiac myxoma as 36 months.^1^ Recent advances in both cardiac and cerebral imaging have eased the diagnostic pathway and increased the frequency of myxoma detection.^2,3^

This article depicts a case study of a female who presented with multiple oncotic cerebral aneurysms caused by an underlying left atrial myxoma. The clinical manifestations and pathogenesis of cardiac myxoma, as well as the imaging pathway, management and prognosis of myxomatous aneurysms will be discussed.

## Case report

An independent, healthy 48-year-old female presented to her general practitioner with a 3-week history of slurred speech, unsteadiness and fatigue. She took occasional iron supplements, but no regular medications.

Her past medical history included iron deficiency anaemia and a melanoma, which had been excised 13 years previously.

One year prior to this presentation she had an admission spanning 4 weeks, with lingering depressive and anxiety symptoms after a presumed viral infection 6 months earlier. She had experienced lethargy, intermittent headaches, nausea, diarrhoea and 16 Kg of unintentional weight loss over a 6-month period. Following investigation with biochemical assessment, endoscopy, colonoscopy and a CT abdomen she was diagnosed with an episode of major depression and started on an antidepressant.

**Figure 1.**
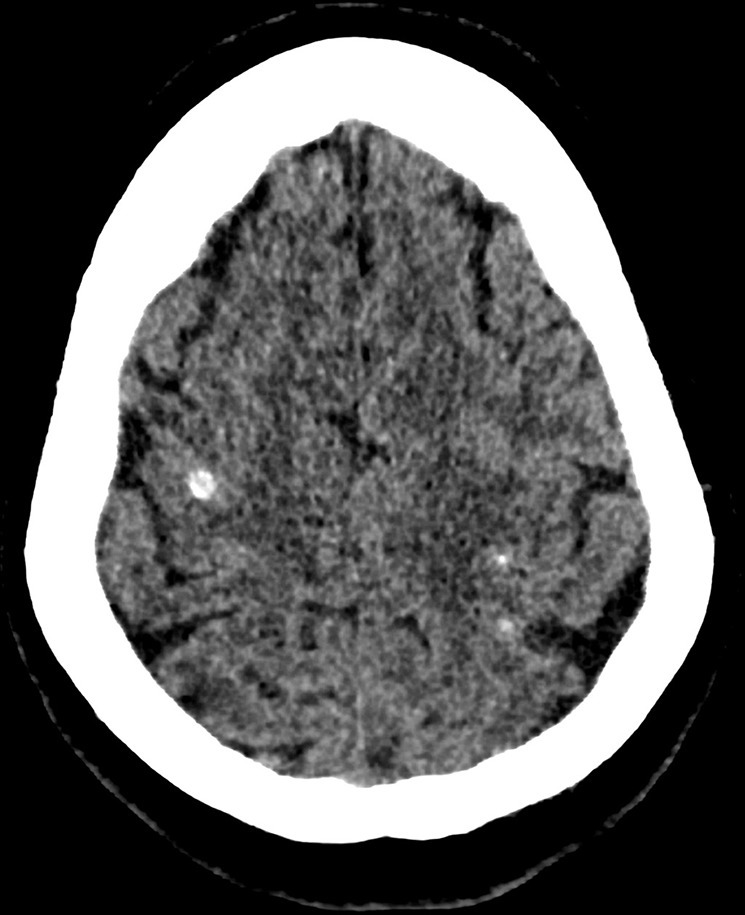
Non-contrast CT brain demonstrating bilateral supratentorial hyperdense punctate haemorrhagic foci. The largest lesion was infratentorial, in the right cerebellar hemisphere (depicted on MRI images).

Clinical examination at this presentation demonstrated ataxia, with the patient being unable to walk heel-to–toe. Romberg’s test was negative. She had a subtle dysarthria. No other focal neurology was elicited on neurologic examination. Cardiorespiratory examinations were unremarkable. The general practitioner arranged a CT brain, which identified multifocal haemorrhagic sites in the right cerebellar and both cerebral hemispheres (Figure 1). These ranged from subcentimetre in size to the largest—a 3-cm right cerebellar lobar bleed. The differential diagnoses postulated in the report were haemorrhagic metastases—possibly recurrent melanoma, or an atypical presentation of amyloid angiopathy.

**Figure 2.**
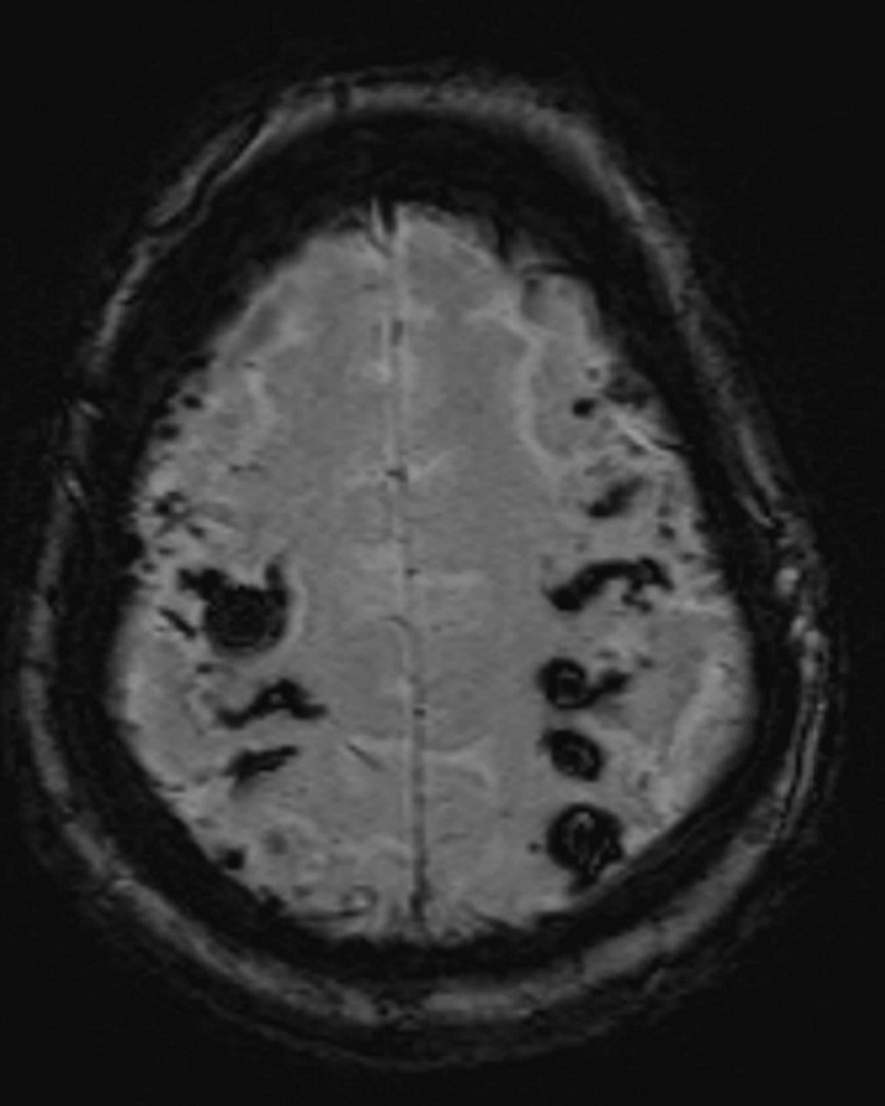
Maximum intensity projection susceptibility weighted image sequence (mIP SWI) MRI demonstrating haemosiderin deposition in the cerebral sulci.

**Figure 3.**
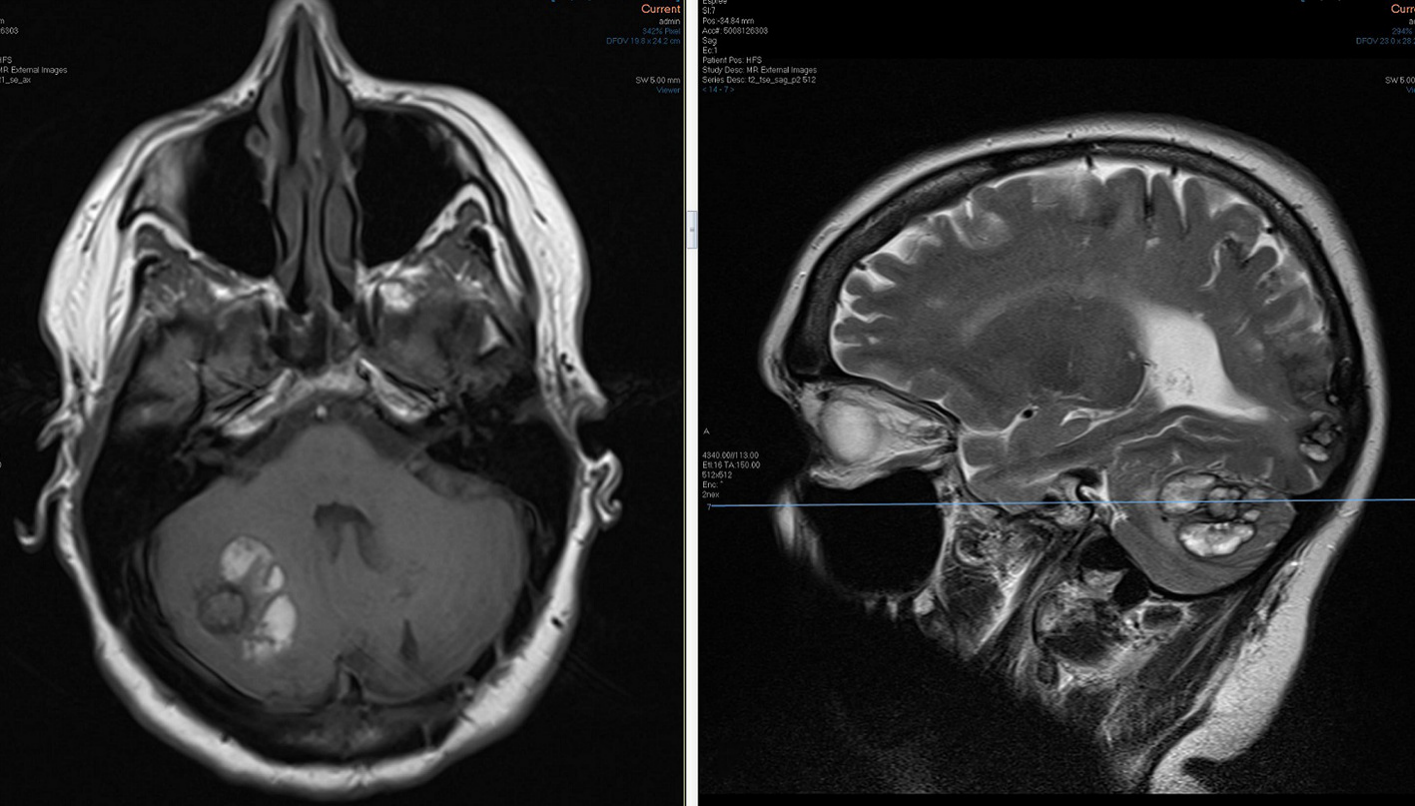
Non-contrast *T*_1_ (left) and *T*_2_ (right) images at the level of an irregular ring- like lesion in the right cerebellar hemisphere. The lesion demonstrates low *T*_1_ signal and high *T*_2_ signal, compatible with a metastasis. Classically a melanoma deposit would demonstrate high *T*_1_ signal. Hyperintense *T*_1_ and *T*_2_ material surrounding the lesion is in keeping with subacute haemorrhage. Note the additional site of haemorrhage in the inferior right occipital lobe, seen on the sagittal slice.

**Figure 4.**
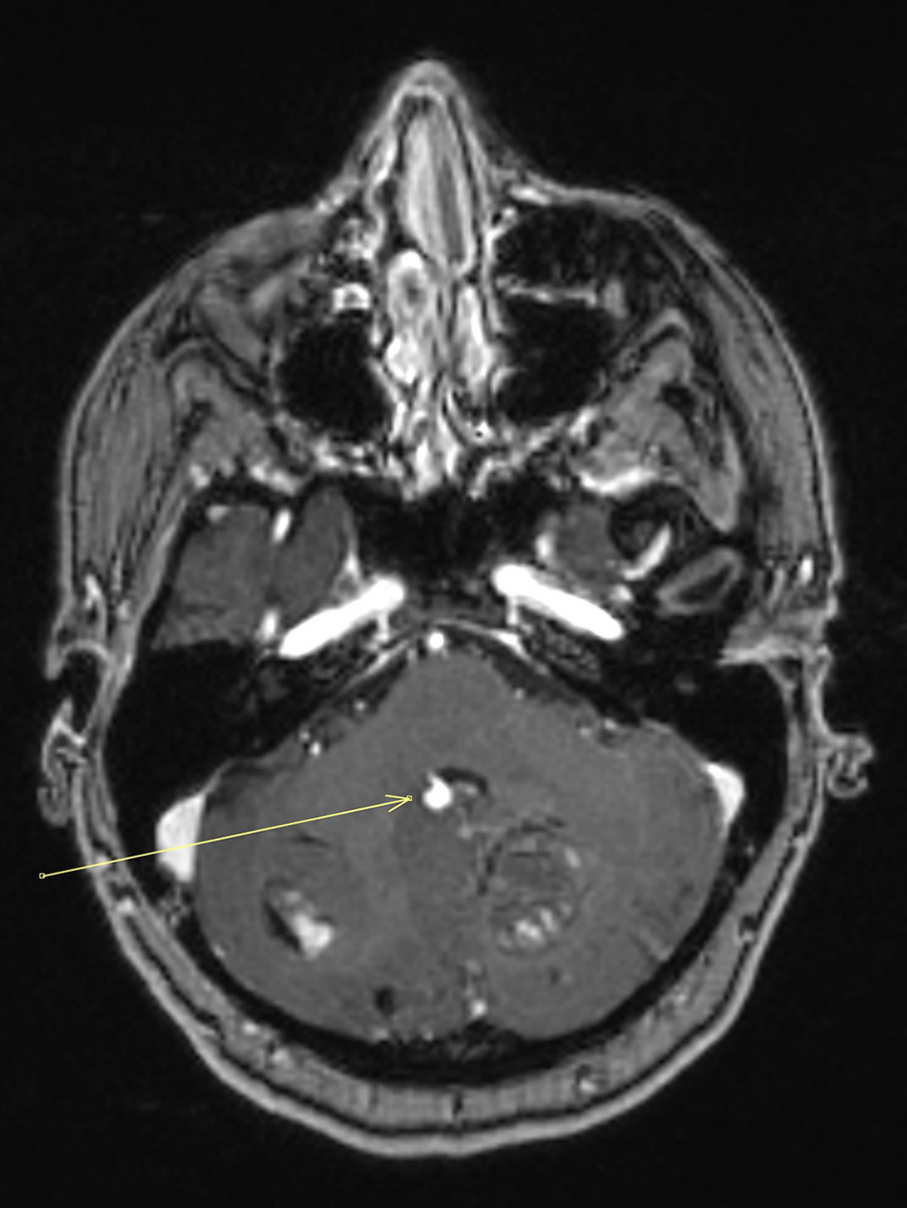
Post contrast *T*_1_ weighted stealth MRI sequence demonstrating bilateral enhancing cerebellar lesions and an avidly enhancing aneurysm arising adjacent the posterior wall of the fourth ventricle (arrow). The lesions are suspicious for metastases.

**Figure 5.**
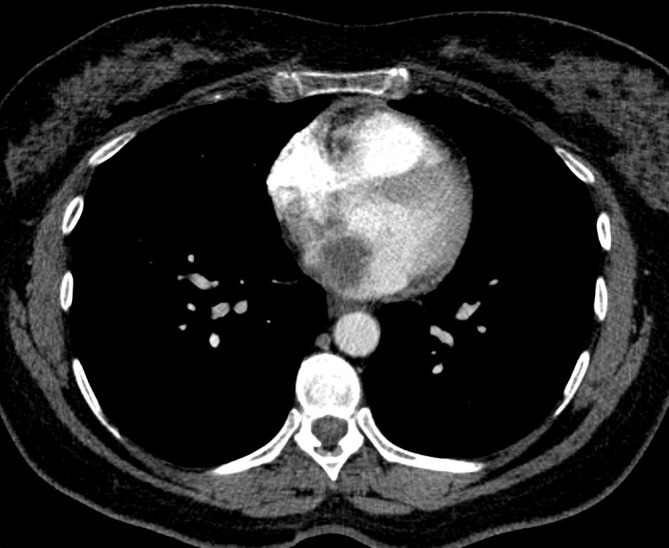
Post contrast, non-gated staging CT Chest showing a left atrial mass, concerning for cardiac myxoma.

The patient was commenced on dexamethasone and referred to the neurosurgical department. She was booked for a staging CT chest/abdomen and pre-operative stealth brain MRI, with a view to excise the right cerebellar lesion for histological diagnosis. The MRI confirmed multiple supra- and infratentorial lesions. Haemosiderin staining was seen in multiple sulci at the vertex in keeping with a low volume of subarachnoid blood, and a 4 mm posterior inferior cerebellar artery aneurysm was detected (Figures 2–4). The CT chest identified a mass in the left atrium concerning for an atrial myxoma (Figure 5). In view of the suspected cardiac mass the neurosurgical biopsy procedure was postponed pending further work up.

Echocardiography showed a large, mobile left atrial mass compatible with an atrial myxoma. There was no associated obstruction despite the mass occupying half of the left atrial volume. Left ventricular function was normal (Ejection Fraction 62%). Without histological support a melanoma deposit remained a possible diagnosis and, after discussion between neurosurgery, cardiology and cardiothoracic teams, a decision was made to proceed with the cerebellar excision. A cardiac MRI was also arranged.

The cerebellar excision was uncomplicated, but histological assessment was non-contributory towards a diagnosis. No melanoma or other malignant cells were identified. The excised material consisted of haemorrhage and some distortion of the underlying vascular architecture, without evidence of vasculitis or amyloid angiopathy. In the interval, the cardiac MRI demonstrated an interatrial septal stalk pathognomonic for atrial myxoma. The new favoured diagnosis was haemorrhagic transformation of embolic ischaemic infarcts secondary to the atrial myxoma.

**Figure 6.**
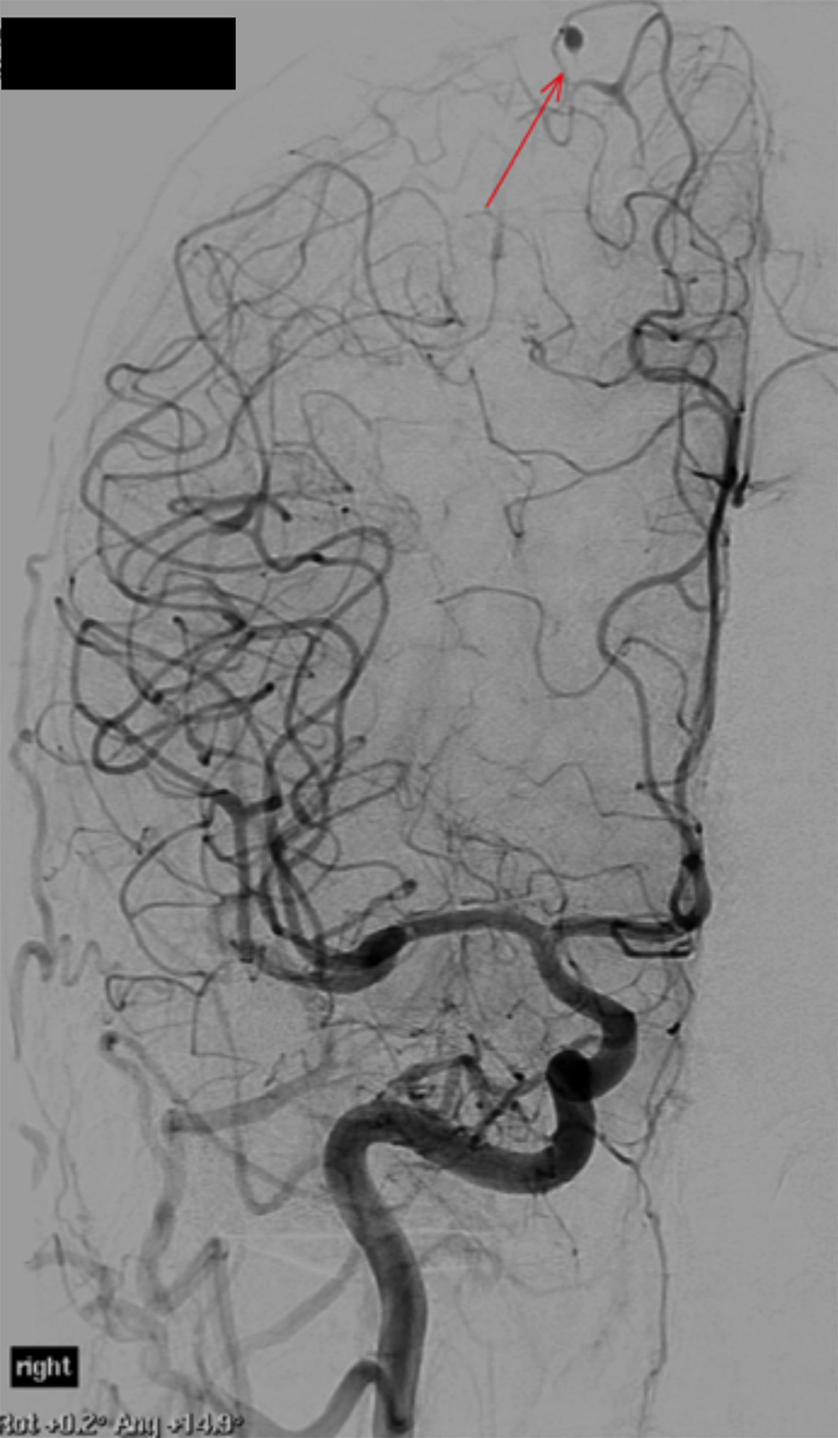
Digital subtraction angiography confirming aneurysm formation. The largest aneurysm is located in a peripheral branch of the right anterior cerebral artery (highlighted by arrow).

**Figure 7.**
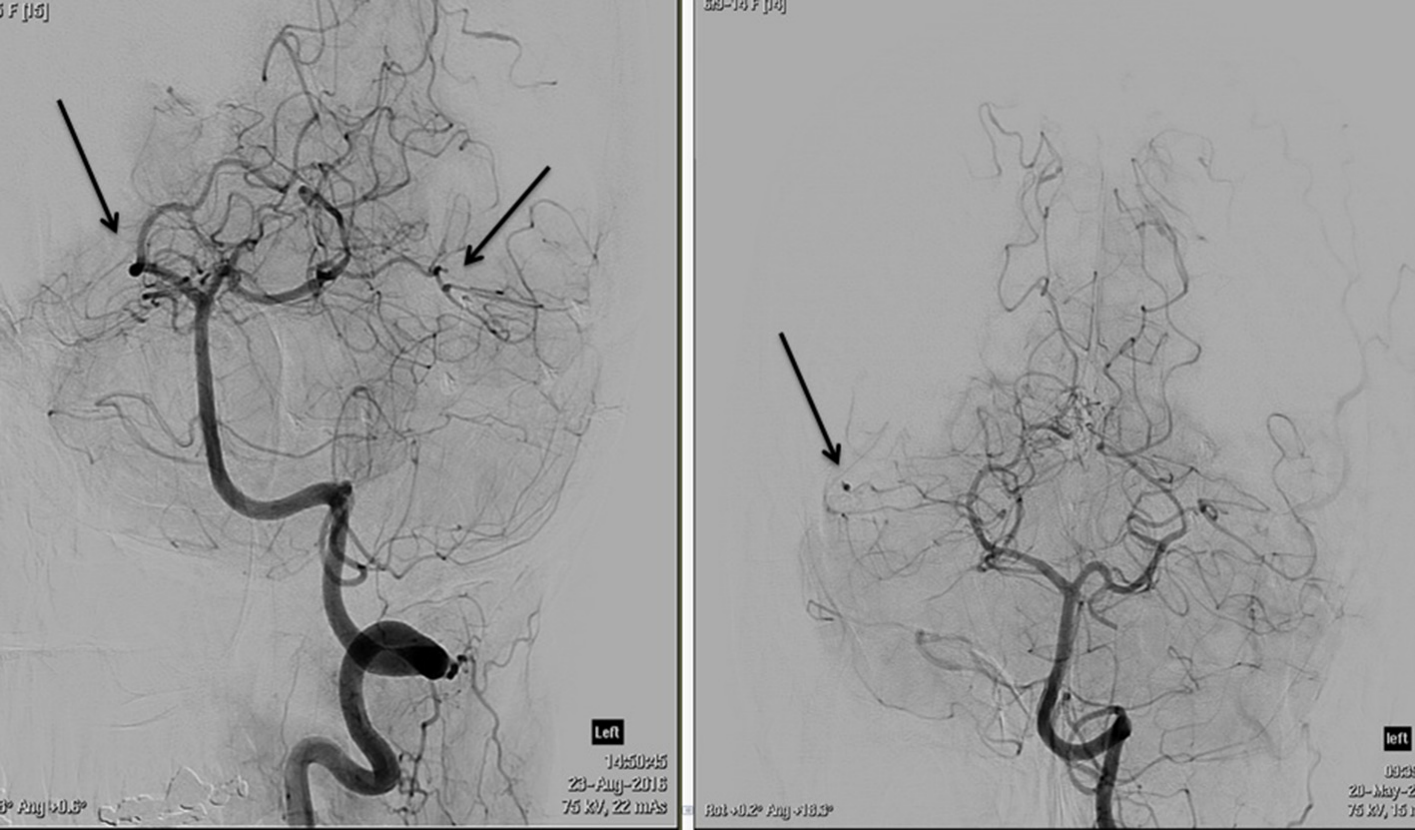
DSA images of the left vertebral artery. There is extensive background fusiform vessel irregularity. The arrows highlight multiple sites of small aneurysm formation.

The patient showed some symptomatic improvement with steroids and was referred to Interventional radiology for formal cerebral angiography assessment to evaluate for cerebral vasculitis. Both internal carotids and vertebral arteries were imaged. The procedure revealed extensive abnormalities, with diffuse irregularity of the medium to small vessels, most pronounced in the peripheral ICA and PCA branches. Distal, fusiform aneurysm formation was identified in at least two distinct sites in the peripheral intracranial branches (Figure 6,7). No central aneurysm was detected around the arterial circle of Willis.

The patient underwent excision of the left atrial mass under therapeutic heparin cover on a cardiopulmonary bypass machine. Histological assessment confirmed a left atrial myxoma, with the friable specimen demonstrating classical stellate mesenchymal tumour cells set in a loose myxoid stroma. The patient had an uncomplicated post-operative course and a normal post-operative echocardiogram. She was discharged home with a follow-up MRI and neurosurgical review booked at 6 weeks.

## General discussion

Cardiac myxomas occur at an incidence of 0.002% in the general population.^4,5^ They are a benign growth originating from multipotential subendocardial mesenchymal cells and are the most common heart tumour, accounting for 50% of all cardiac tumours.^5,6^ Most cases have been reported in the 40- to 80-year-old age group. There is debate in the literature over whether there is a female predominance.^5^ 75% are located in the left atrium and this site is most commonly associated with neurological complications.^5^

Atrial myxomas classically manifest with a clinical triad of constitutional symptoms, cardiac dysfunction and embolic phenomena.^5,6^ Constitutional symptoms are often non-specific and can therefore be misleading. Fever, arthralgia, myalgia, rash and cachexia all occur commonly and can direct clinical suspicion towards infection, systemic inflammatory rheumatologic processes, or disseminated malignancy.^5,6^ Accompanying laboratory findings can also be non-specific and include leucocytosis, elevated C-reactive protein and erythrocyte sedimentation rate, and/or derangement of liver transaminases (hypothesised to be secondary to cardiohepatic congestion).^5^ Cardiac effects are usually due to mechanical obstruction of the chamber or valves but can manifest with arrhythmia, or myocardial dysfunction secondary to direct tumoural infiltration of the myocardium.^5^ Embolic phenomena are systemic or neurologic, and usually precede cardiac failure in the clinical presentation of cardiac myxoma.^4–6^ A patient presenting with isolated or recurrent embolic events should prompt further assessment with cardiologic examination and non- invasive cardiac imaging to exclude a cardioembolic source.

Cardiac myxomas are associated with a spectrum of cerebral complications.^4–8^ The most frequent neurological complication is acute cerebral ischaemia secondary to embolic tumour material causing vascular occlusion.^4–7^ Up to 50% of patients with atrial myxoma initially present with stroke symptoms.^5,7^ Embolisation instigating progressive cerebral vascular stenosis or aneurysm formation is less common. Other rare neurologic complications include parenchymal metastases and intracerebral haemorrhage secondary to rupture of a cerebral aneurysm.^5,8^

The true incidence of aneurysms secondary to cardiac myxoma is unknown, with current evidence determined by less than 60 case studies. The pathogenesis of aneurysm formation is also not well known.^5,9^ Embolisation of tumour particles to the cerebral vessel walls is favoured to be the predominant mechanism for aneurysm formation, with the embolised myxoma cells thought to induce aneurysm formation by penetrating and weakening the vascular walls.^4–6,9^

Some studies have contradicted this hypothesis with description of new cerebral aneurysms that form many years after myxoma resection.^1,7^ Reports of persistently elevated serum Interleukin-6 (IL- 6) levels suggest that the pro-inflammatory effects of IL-6 may induce or contribute to aneurysm formation in a paracrine manner in some patients.^9^ Atrial myxoma cells have been shown to produce IL-6 and in some patients, including some with new aneurysm formation post myxoma resection, persistently elevated serum IL-6 levels have been identified.^6,9^ IL-6 has been postulated as a potential seromarker to predict patients at risk of delayed complications, but further research is required.

## The role of imaging in the diagnostic pathway

Transthoracic echocardiography is the first step in detection and imaging characterization of intracardiac tumours. It is non-invasive, relatively inexpensive and can identify the location, size and mobility of the mass.^2,5^ Mobility characteristics can be used to distinguish atrial myxoma from left atrial thrombus, with myxoma more likely to be mobile and demonstrate an attachment stalk.^5,10^ Transoeseophageal echocardiography is more invasive, but can detect smaller mass lesions and offers the benefit of more accurate assessment of atrial appendages and extracardiac extension.^2,3,5^ In addition to detection of an intracardiac mass, echocardiography is used to quantify underlying or secondary haemodynamic effects such as reduction in ejection fraction and valvular dysfunction.

Cardiac CT is a valuable adjunctive technique, increasingly being used in the pre-operative work up of intracardiac tumours.^3,5,6^ Cardiac CT can demonstrate fat and calcification within the mass, and can delineate both coronary and tumour blood supply.^5^ Although echocardiography is often the initial imaging modality, gated cardiac MRI has become an indispensable tool for accurate delineation of the tissue characteristics and functional impact of a cardiac tumour. Cardiac MRI is also the gold standard for demonstrating intra and extracardiac extension, including involvement of the mediastinum and great vessels.^5,11^ It therefore plays an important role in surgical planning.

The standard protocol of a cardiac tumour MRI is designed to answer the four main objectives of the study, namely, location and extent of tumour(s), characterization of intrinsic tumour tissue, quantification of inflow and outflow tract obstruction, and background ventricular volume and function.

**Figure 8.**
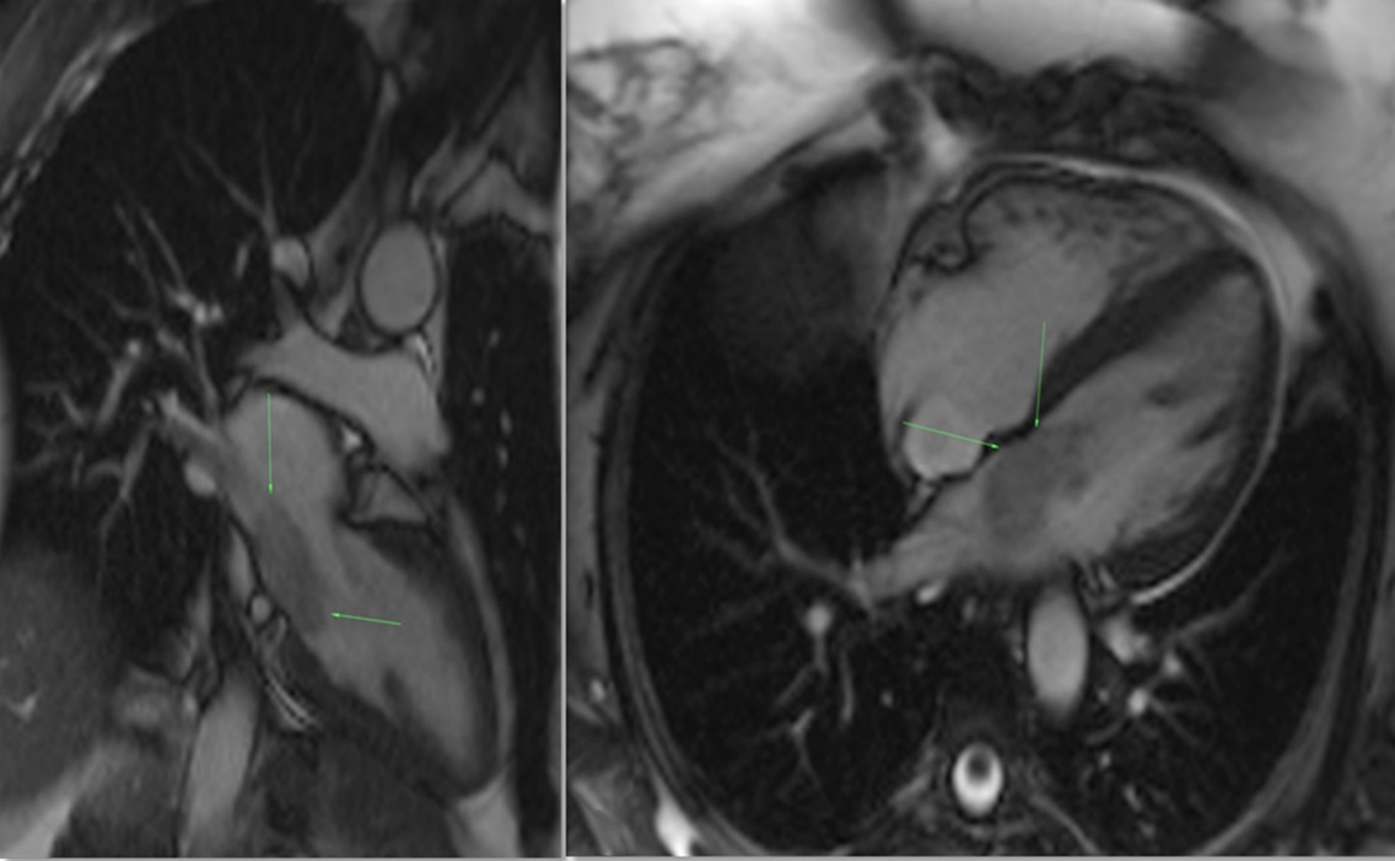
Cine SSFP 2-chamber and 4-chamber views demonstrating the left atrial myxoma (see arrows), which is hyperintense relative to the myocardium and hypointense relative to the blood pool. The attachment stalk, difficult to appreciate on a static image, becomes apparent with cardiac motion. SSFP, Steady-state free procession.

Steady-state free procession (SSFP) cine sequences in 2- and/or 4-chamber planes address the location, gross morphology including attachment and functional impact of a myxoma. Employing ultrafast gradient echo sequences, and obtained with cardiac gating and multiple breath holds, cine SSFP imaging can both distinguish the myxoma from the adjacent blood and myocardium, and highlight its dynamic obstruction to blood flow.^12^ Typically the myocardium, myxoma and blood are distinct from each other, in an order of increasing signal intensity. During the course of the cardiac cycle the attachment stalk becomes apparent as the tumour moves, and potentially prolapses, through the atrioventricular valve (Figure 8).^12^ In comparison with an atrial myxoma, malignant tumours are less mobile, often larger in size, display a broad attachment base, extend into multiple cardiac chambers and/or more readily demonstrate extra-cardiac involvement.^5,11^

**Figure 9.**
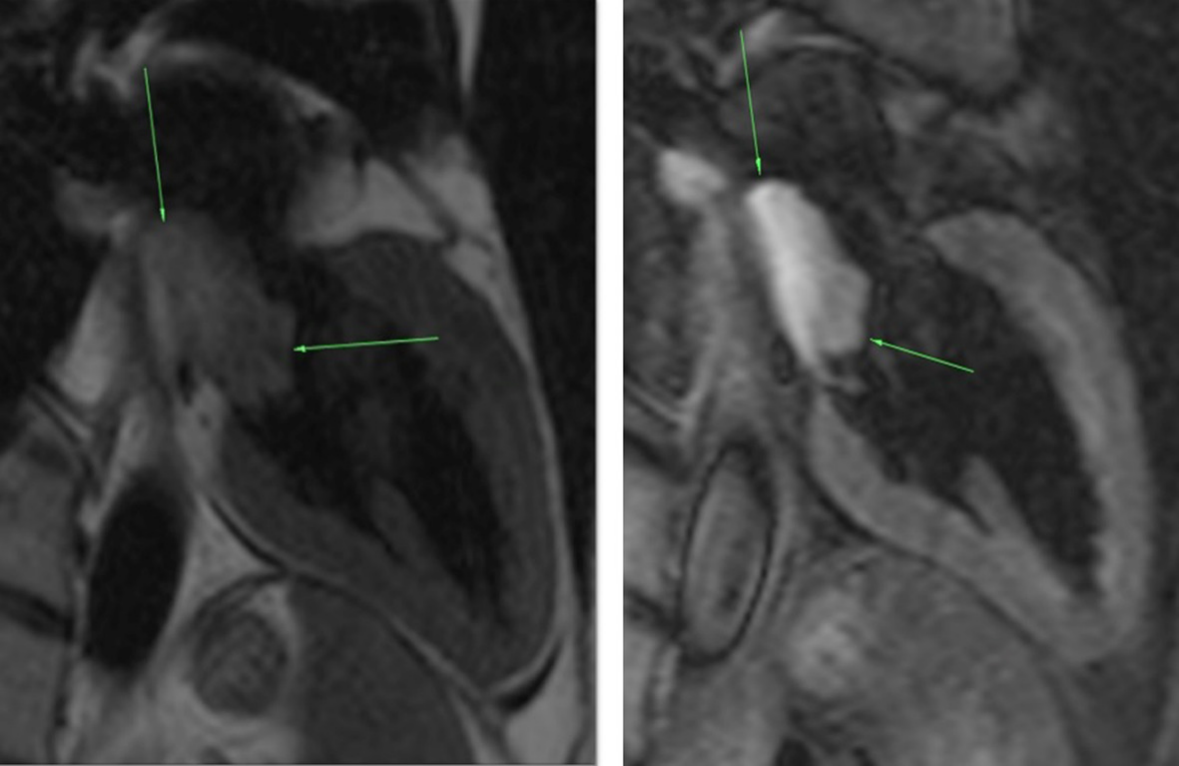
Dark Blood *T*_1_ (left) and *T*_2_ fat-suppressed (right) 2- chamber sequences demonstrate intermediate signal intensity, and hyperintensity, of the myxoma respectively (myxoma highlighted by arrows).

*T*_1_, *T*_2_ and/or *T*_2_ FS weighted dark blood ultrafast spin echo sequences, oriented in perpendicular planes to the mass, are used for characterization of intrinsic tumour tissue. Normally, a myxoma will demonstrate intermediate *T*_1_ signal and heterogeneous hyperintensity on *T*_2_ weighted images (Figure 9).^12^ This reflects the myxoid, fibrous and blood product constituents. Internal calcification (often well demonstrated on CT) will correspond with areas of low *T*_1_ and *T*_2_ signal. These signal characteristics are in contrast to an intracardiac thrombus which is generally homogenously hypointense on *T*_1_ and *T*_2_ and, relevant to this case, a melanoma metastasis which would classically demonstrate hyperintensity on both *T*_1_ and *T*_2_ imaging, due to its intrinsic melanin content (Table 1).

**Table 1.** Comparative table to highlight the MRI characteristics of myxoma, thrombus and melanoma—the three main differential diagnoses in this case

	Myxoma	Thrombus	Melanoma
Location	Left atrium at atrial septum, near fossa ovalis	Left atrial appendage, left atrium, or in association with hypokinetic infarcted myocardium	Epicardium commonly invaded, LV free wall and LV septum. Associated pleural +/- pericardial disease or effusion.
Morphology	Circumscribed, narrow attachment stalk (pedunculated)	Broad based	Broad base, infiltrative with irregular width and heterogeneity of LV wall
Mobility	Highly mobile, can prolapse through valve	Less mobile	Less mobile
Intensity	*T*_1_ intermediate, *T*_2_ heterogeneously hyperintense	*T*_1_ hypointense *T*_2_ hypointense homogenous	*T*_1_ hyperintense *T*_2_ hyperintense heterogeneous
Enhancement	Heterogeneous	Uncommon, unless peripheral fibrous tissue present (usually chronic thrombus)	Heterogeneous

**Figure 10.**
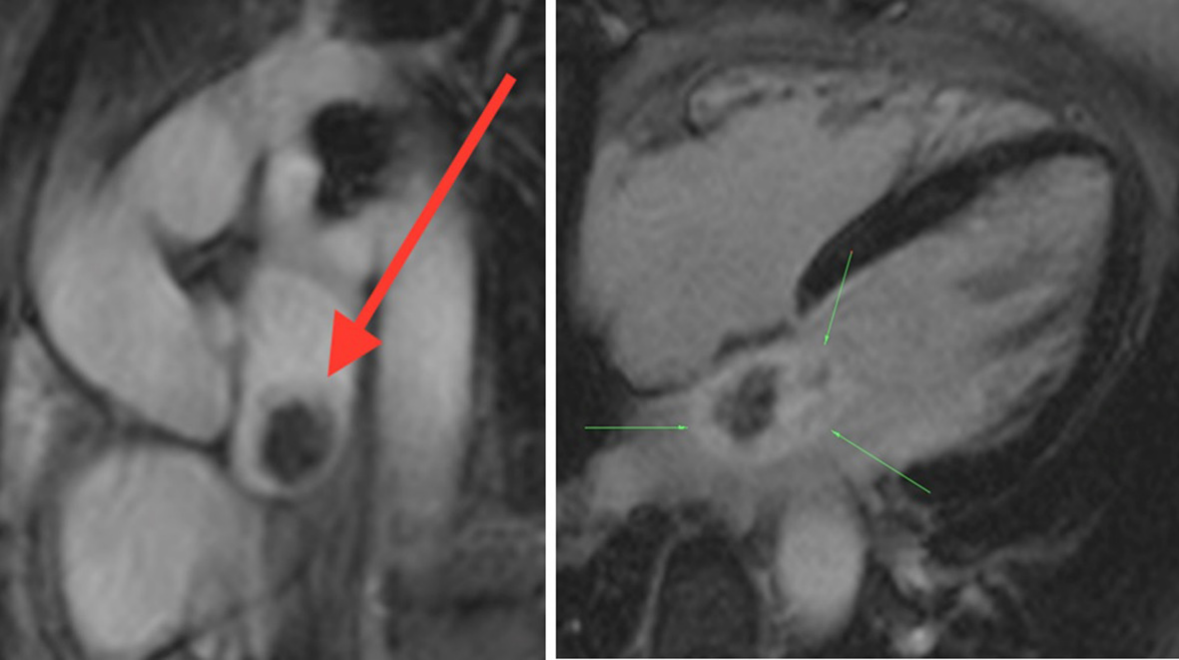
First pass perfusion imaging (left) allows bright gadolinium contrast to clearly outline the mass. Delayed contrast 4-chamber view (right) shows heterogeneous enhancement of the mass (see arrows). The internal non-enhancing area likely corresponds with a region of necrosis.

When echocardiography is equivocal, dynamic contrast enhancement sequences can definitively distinguish a tumour from the most common cause of an intracardiac mass—a thrombus.^5^ Contrast-enhanced sequences are taken with first pass perfusion, and at a 10 to 15-min delay to confirm late gadolinium tumour enhancement. While a thrombus does not enhance, or shows only minimal peripheral enhancement in fibrous elements, a myxoma shows definite heterogeneous enhancement on the delayed sequences (Figure 10). It may show mild heterogeneous enhancement on the first pass perfusion. Histologically, areas of poor enhancement correspond with internal areas of necrosis.^12^

Neuroimaging with CT and MRI has identified embolic strokes in all vascular territories.^5–7^ The insults can be ischaemic, or haemorrhagic, with lobar and subarachnoid haemorrhage observed in association with rupture of an oncotic aneurysm.^5,6,13^ CT and MR angiography are often used to outline the distribution of the aneurysms; however diagnostic cerebral angiography remains the study of choice for detection of these lesions.^5,13^ The ability to identify aneurysms on a delayed contrast washout phase improves sensitivity of angiography in detecting aneurysms, above cerebral CT and MRI. The aneurysms occur most commonly in small peripheral branches of the anterior and middle cerebral arteries, and are typically fusiform in shape.^5,6^ Saccular aneurysms are not as common.^7^ Extracerebral arterial aneurysm formation has been described in association with intracardiac tumours, but is very rare.^5^

## Treatment and prognosis

Excision of the cardiac mass is mandatory both for symptomatic improvement and to prevent further embolic complications. The operative mortality for atrial myxoma resection is less than 2%.^14^

Resolution of constitutional symptoms, cardiac impairment and biochemical abnormalities is expected following surgical resection.^5,7,15^ There are also observed cases of oncotic aneurysm regression post myxoma resection.^6^ Conversely, there are reports of delayed cerebral aneurysm formation, even many years after myxoma resection. Further study is required to elucidate which patients are at risk of delayed cerebral complications. Treatment of neurologic complications of atrial myxoma is uncertain, with the strength of evidence limited to case reports and case series. Thrombolytic therapy for ischaemic emboli has brought mixed results, with prior research suggesting that success of thrombolytic therapy is likely dependent on two factors; the composition of embolic material, and the presence or absence of oncotic aneurysms.^5,6^

Treatment of aneurysms is also unclear and, with most of the aneurysms fusiform in shape, their amenability to clipping or coiling can be limited. While case reports of endovascular coiling for enlarging aneurysms have described success, overall evidence documenting stability of cerebral aneurysms over several years gives support to conservative treatment management options.^4–15^

## Conclusions

Our case report highlights several important features of the diagnostic and treatment pathway for atrial myxoma—in particular, the potentially nonspecific clinical presentation and oft-prolonged time to diagnosis. Multidisciplinary communication is critical in seeking the unifying diagnosis, and the use of multimodality imaging allows accurate characterization of both the primary tumour and extent of its systemic complications. Our patient, after presenting with oncotic aneurysms complicated by haemorrhage, will have ongoing cerebral MRI monitoring to assess the size and morphology of the residual aneurysms. Imaging frequency will be guided by the patient’s ongoing symptoms. Five years of semi-annual transthoracic echocardiography surveillance has been recommended to screen for local recurrence, which occurs at an incidence of 3% in cases of sporadic myxoma and is highest in the initial 4 years post operation.^16^ There are no clear treatment guidelines with regard to management of perseverant oncotic aneurysms and therapy decisions will be guided by the stability of imaging findings.

## Learning points

1. Isolated or recurrent embolic events should prompt further work up with cardiac examination and echocardiography.
2. Cardiac myxoma can result in a spectrum of cerebral and systemic vascular complications, ranging from infarction, to oncotic aneurysm formation and parenchymal metastases.
3. Cardiac MRI is the modality of choice for diagnosing myxoma, delineating the extent of local growth, and quantifying its functional impact.
4. Delayed cerebral oncotic aneurysm formation does occur post cardiac myxoma resection, and may warrant surveillance cerebral imaging.
5. Long-term management of oncotic cerebral aneurysms should be guided by the clinical context, aneurysm morphology and stability of imaging findings.

## Consent

Written informed consent for the case to be published (including images, case history and data) was obtained from the patient(s) for publication of this case report, including accompanying images.
